# Manual of Physiology

**Published:** 1849-09

**Authors:** 


### REVIEWS.

---

ART. V.—*Manual of Physiology*. By W. SENHOUSE KIRKES, M. D. Assisted by JAMES PAGET, Lecturer on General Anatomy and Physiology, at St. Bartholomew's Hospital, with 118 illustrations on wood. Philadelphia: Lea & Blanchard, pp. 552, 12mo. 1849.

That branch of medical science in which progress for some years past has been most rapid, is unquestionably Physiology. Whatever may be said of the advances of practical medicine, the most cynical are obliged to admit that physiology has been enriched by great discoveries. The student of former years, indeed, who returns to it now, finds it a new science. It is no longer what it was when Blumenbach and Rickerand were the only authorities accessible to American students in their mother tongue. It has not only been extended by novel and important accessions, but has assumed a different aspect.—It has become an exact science. Based upon anatomy, and growing out of it, it has ceased to be visionary, and has grown eminently practical. Chemistry and the microscope have explained functions concerning which, but a little while ago, we had nothing but ingenious surmisings. Many absurd theories have been abandoned, and, what is of far greater account, better processes have been adopted. It is not by a resort to some imaginary vital force, but to the scalpel, the chemical reagent, or the microscope, that the physiologist seeks to solve the difficult problems of his science. Speculation is given up as of no value. We do not find the word "sympathy," which held so conspicuous a place in some exploded systems, once mentioned in this latest treatise on physiology.—Mesmerism, if mentioned at all, is alluded to incidentally —indications, these, to our minds, of real advancement in physiological science.

Human physiology is defined by our authors to be "the science which treats of the conditions, phenomena, and laws of the life of the human body in the state of health." These phenomena are such as are exhibited by vegetables, in common with animals, and such as are peculiar to the members of the animal kingdom. Of the first class are the processes of digestion, absorption, secretion, circulation, respiration, and some others, the processes also of growth and nutrition, and the generative process.—These are the phenomena of organic or vegetative life.—Those which are peculiar to animal life are the functions of sensation and voluntary motion. But it must be borne in mind that this division of the functions of life is artificial, and does not indicate any absolute dissociation, the organic and the animal life being so knit together and so mutually dependent, that neither can be long maintained without the other. If the processes of digestion, respiration, and nutrition are essential to the maintenance of the organs of animal life, sensation and voluntary motion are not less so to the prehension of food, and other functions of organic life, by which the animal is kept alive and the species perpetuated.

Organization is peculiar to the objects of the living world. All bodies in which the phenomena of life have been observed are formed of diverse mutually adapted parts, which are styled organs; and it is nowhere else but in such bodies that organization is detected. We find it in the fossil, but not in the quartz pebble. The fossil, indeed, may be silicified and crystalized, but in the silex and amid the crystals the impress of its organs is still palpably manifest, its eyes, its stomach, its weapons of defence hardened into flint.

The first chapter of this work treats of the chemical composition of the human body, into which about one-third of the elements with which chemistry has made us acquainted are now known to enter. Four of the constituents, to wit: oxygen, hydrogen, nitrogen, and carbon, because of their uniform existence and great abundance in nearly all animal substances, are named *essential elements;* the rest, being less constant, and occurring often in very small quantity, are styled *incidental elements.* But we are not to infer that these are less necessary than the first class to the right composition of the substances in which they exist. Sulphur and phosphorus are as essential to the constitution of the tissues, and iron is as essential to the constitution of the blood, as is either of the four abounding elements. The terms are to be taken in only a general sense. The existence of manganese, alumina, and copper in the human body has been questioned —but the experiments of Orfila, Millon, Hiller and others leave little room for doubting the fact that they often occur. Alumina is found in the ashes of hair, bones, and enamel, and manganese and copper exist especially in the biliary secretion. In the blood as well as the liver of several of the mollusca and fish, Harless and V. Bibra found copper constantly present, seeming to replace, to some extent, the iron contained in the blood of other species, and to form a normal, necessary constituent. Still, in the view of our authors, it is most likely that, in the human body, both copper, manganese and alumina are "*accidental elements*, which, being taken in minute quantities with the food, and not excreted at once with the feces, are absorbed and deposited in some tissue or organ, of which, however, they form no necessary part." Arsenic and lead, in the same manner, being absorbed, are occasionally deposited in the liver and other parts. In favor of this view, we have the statement of Heller, that although copper is frequently present in the bile of adults yet it is never found in that of infants.

It would be curious to compare the statements contained in this chapter with the teachings of a school of physiologists once somewhat in vogue. Few contrasts could be found more striking. By these physiologists it was held, that neither the essential nor the accidental elements extend, as such, into the composition of the human body —that neither carbon, oxygen, nor hydrogen, neither iron, phosphorus, nor sulphur, could be found existing "formally" in animal matter. They held that the chyle was a "homogenous fluid," unvarying in its constitution, and that it alone entered the circulation. Neither lime, nor soda, nor any other foreign substance, they contended, could pass the lacteals, but that these matters were formed by the "vital principle" out of the elements of the food. Sulphur and phosphorus, if taken up by the lacteals, were absorbed as "chyle," and only reappeared as sulphur and phosphorus when they were thrown off as excretions and placed beyond the pale of vitality. When a deficiency of phosphate of lime was at any time manifest in the system, the deficiency was ascribed, not to any defect in the food, but to a lack of energy in the vital principle. Digestion was impaired, and the phosphorus, oxygen, and lime could not be created. Out of the "homogenous chyle" the phosphoric acid and calcium of the bones, the chlorine, and sodium, and potassium of the blood, the iron of the hematozine, the magnesia, silica, fluorine, and manganese, all were held to be created by the vital principle. The same chyle formed, in one organ, bile, and in another, urea; in one it was deposited as fat, and in another it repaired the wasted muscular or nervous tissue. The "homogenous chyle" gave birth to the "homogenous blood," and out of this simple fluid sprang compounds of endless variety.

One reads these lucubrations now with a smile. No one thinks of opposing them with serious argument.—The system, if system it is worthy to be called, "like the baseless fabric of a vision," has faded away, and soon no wreck of it will be found in our medical literature.

The second chapter gives an account of the structural composition of the human body. Fluids and solids compose it. The fluids are, 1st, *formative*, from which are derived the materials for the construction of the frame ; and, 2d, *secreted* fluids.

Among the solids, some are structureless, as the albumen of eggs, the substance called *cytoblastema*, or *formative* substance, the simple membrane which forms the wall of most primary cells, the membrane enveloping the vitreous humor of the eye, and the intercellular substance of the most perfect cartilage. In those which present determinate structure, certain primary forms may be distinguished, which make up the tissues and organs of the body. These are, 1st, *granules*, or *molecules*, the simplest and minutest of the primary forms ; 2d, *nuclei*, or *cytoblasts*, which stand next in simplicity of structure ; 3d, *cells*, larger than nuclei, but composed, like them, of membranous cell-walls, with, usually, liquid contents.—By the further development of these, higher, or secondary forms are produced ; as, 1st, *filaments*, or fibrils ; 2d, *fibres;* 3d, *tubules.* These form the various tissues of the body, which are briefly described in the subsequent chapters of this work.

The third chapter considers the vital properties of the organs and tissues of the human body ; for while chemical and mechanical forces are sufficient to account for many of the phenomena of living beings, there are others which cannot be explained upon this principle, and which imply the existence of another force, the vital. A remarkable manifestation of this force is seen in that property of vegetables and animals, by which they form themselves out of materials unlike them ; as when, for example, a plant grows by assimilating the elements of ammonia, carbonic acid, and water ; or when an animal feeds upon vegetables and forms its blood and various organs out of the materials of its food. This is termed *formative*, or *plastic force*, which exhibits itself under the three forms of *development*, *growth*, and *assimilation*. The most general, and one of the simplest, modes in which this force is manifested is in the formation and further development of nucleated cells.

The nucleated cell is the form towards which organizing matter most commonly tends, in which it often rests, and through which it usually proceeds in its further developments. In nucleated cells, also, are the best examples of inherent formative power, which, not being consumed in the formation of the cells, remains operative in them, changing them and their contents, and influencing the surrounding or intercellular substance in which they are deposited. Thus, whether it be for the preparation of materials from food which may serve to the maintenance of the body, or for the construction of the several tissues, or for the formation or temporary storing up of the materials that are to be removed from the body as refuse, in all these, and in nearly all instances of them, the end is attained by or with the help of the formation, continued energy, or dissolution of nucleated cells.

It is no single, but several complex forces which accomplish the formation of so simple a part as a cell.— Mechanical force determines its position, shape and relations ; chemical force decides the composition of its walls and contents ; but, co-operating with these, and directing them, is the wonderful vital force, " by virtue of which the cell acquires all the properties that characterize the species or organ to which it is attached, and becomes capable of taking part in vital processes—even in such processes as those in which itself originated."

Some physiologists have represented these forces as in a state of antagonism. Chemistry, they teach, was at war with vitality, and only gains any influence over the body when life is at an end. But such is not the opinion of our authors, who teach that "the vital formative force includes and directs the physical forces that issue from the mere matter of the organic body." How much in each act of the living body is physical, and how mnch depends on the peculiar vital force, is more than we can determine ; but Dr. Kirkes truly remarks that "the advancing knowledge of the physical sciences proves every year that effects which used to be ascribed to vital forces, are due to the operation of the forces of chemistry and mechanics."

Contractility may be reckoned a second vital property, and a third is the power of conducting or transmitting stimuli or impressions ; and these three are the peculiar properties of living animal matter. Self-formation, which every part possesses, constitutes, in its various modes of action, the principal functions of the organic life of the animal. In some of the tissues resides the power of contracting ; and the nervous tissue is capable of peculiarly conducting the impressions made on it by stimuli. The latter two are exercised especially in the animal life, and it is by means of these that the mind becomes cognizant of the properties of the external world.

The processes of digestion, absorption, respiration, secretion, and exeretion have their end in the formation, movement and purification of the blood. The physiology of the blood, therefore, properly precedes the consideration of these subservient processes. It is discussed at considerable length in the 4th chapter.

The blood is described as "a colorless fluid, containing minute particles, the greater part of which are red, and give the blood its color." The fluid is the *liquor sanguinis;* the particles are the *blood-and-lymph- corpuscles,* or *cells.*

The specific gravity of the blood is 1055, water being 1000, and the extremes consistent with health being, according to Nasse, 1050 and 1059. It has a slight alkaline reaction; a temperature of 100 degrees Fahrenheit; "and emits an odor similar to that which issues from the skin or breath of the animal from which it flows." The alkaline reaction is a constant character of the blood in all animals and under all circumstances, the menstrual blood forming no exception; for the acid reaction which this sometimes presents, has been ascertained to depend upon an acid mucus from the uterus and vagina. Pure menstrual blood, such as may be procured by means of a speculum, is always alkaline, and resembles ordinary blood. The odor of the blood depends upon the presence of a volatile fatty acid, and when sulphuric acid is added it evolves the odor, by combining with the base with which, naturally, this fat is neutralized. This effect is produced by the acid long after the blood has lost the odor which it emits when fresh drawn; and Barruel has directed attention to the curious fact that the likeness of the odor to that of the body of the species of domestic animal from which any specimen of blood has been taken, makes it easy to determine the species. The strong odor of the pig or cat, and the peculiar milky smell of the cow are said to be especially easy to be discerned in their blood.

Of the several constituents of the blood, the fibrine is the only spontaneously coagulable material. Why it coagulates is not certainly known, but the most reasonable hypothesis is, "that its coagulation is a process of organization—the first step towards a more highly organized tissue. The evidence for this hypothesis is given at some length, and is entirely satisfactory, justifying the expression of Mr. Hunter, that "coagulation is an operation of life."

Rest is favorable to the coagulation of the blood, and cold retards it; but of all external conditions that which favors coagulation most, is the free access of air. This has been accounted for by the fact that carbonic acid is extracted more readily under such circumstances; but the explanation is not free from objections, for Dr. John Davy has shown that this gas is not evolved from coagulating blood. Coagulation is not always prevented by the modes of death noticed by Mr. Hunter, although in many instances of death from lightning it has been found fluid in the body. The result does not appear to be uniform.

The blood corpuscles are red and white, the former in the human subject being about fifty times more numerous than the latter, while the individual is in health; in certain diseases the proportion of white corpuscles is as high as one to ten. As a whole, the composition of the blood is so nearly identical with that of the flesh of an animal that the same formula will answer for both, which, as given by Playfair and Boeekmann, is $C_{45}$, $H_{39}$, $O_6$, $N_{15}$.—Its several constituents are subject to great variations.—The water may vary every hour, according to the food and drink, the exercise, the state of the atmosphere, and whatever other events affect the ingestion or excretion of fluids. Its healthy range is from 700 to 790 in the thousand; its average proportion being 784. The albumen may vary, consistently with health, from 60 to 70 parts in the thousand of blood; and the fibrine may vary between 2 and 3 parts. In disease the variation is much greater.

Hemato-globulin is the name given to the substance in the red blood-corpuscles, of which its hematine is remarkable for the large proportion of iron contained in it. The metal, in the opinion of Scherer and Mulder, is combined as an element with the four essential elements of the hematine, as sulphur is combined with them in albumen and fibrine. This view is supported by the fact that when chlorine, which would not decompose an oxide of iron, is passed through a solution of hematine, chloride of iron is formed; and that all the iron may be removed by sulphuric acid, without abstracting from it any of its oxygen, which would not be possible if the iron were more intimately united with the oxygen than with the other elements of the hematine. It is further sustained by this, that hematine loses none of its iron by being subjected for several days to the action of dilute hydrochloric or sulphuric acid, while these acids readily dissolve an oxide and decompose a carbonate of iron. But the color of the blood, it is now rendered certain, depends less upon the iron than on the other constituents of the hematine; for Mulder and Scherer have shown that hematine retains its characteristic hue after all its iron has been extracted. The change of color induced by respiration would seem to depend upon physical rather than upon chemical causes; to be due, in a word, to changes in the form of the blood-corpuscles, and their consequently different modes of reflecting and transmitting light. Black blood becomes scarlet by washing it with salt; a scarlet clot is made black by washing it with distilled water; but these changes are not produced by salt when added to hematine, and are not connected with any chemical change in that substance or in the corpuscles. They are connected with alterations in the shape of the red corpuscles, which are rendered deeply bi-concave, by dense saline solutions, and thickly bi-convex or spherical, by distilled water, changes corresponding with which are produced by the contact of oxygen and of carbonic acid with the corpuscles—the former contracting them, and making their cell-membranes thick and granular; the latter dilating them, and finally dissolving their cell-walls. "Herein, then," as our authors conclude, "is a sufficient explanation of the changes that the corpuscles undergo, without supposing any immediate chemical alteration in the hematine; an alteration which should take place as well in a solution of hematine as in the corpuscles."

The inorganic constituents of the blood, those which form the ashes after its complete burning, are small in proportion to its animal constituents. Of these, the phosphate and carbonate of soda, and the phosphate of lime possess peculiar interest. To the salts of soda the blood probably owes its alkaline reaction. Liebig and Enderlin ascribe it entirely to the tribasic phosphate of soda, and contend that no carbonate is present, for the reason that carbonic acid is not evolved when hydrochloric acid is added to a concentrated solution of the alkaline salts of the blood. But this is explained by the fact, as pointed out by Marchand, Lehmann, and others, that the carbonic acid is absorbed by the solution of the phosphate of soda, and cannot appear as gas escaping. The latest researches of Lehmann seem to have proved the existence of both the carbonate and tribasic phosphate in the blood, and that they are jointly the source of its alkaline character. Liebig has pointed out the remarkable capacity of solutions of phosphate of soda for absorbing carbonic acid, and the readiness with which they give it off again when agitated with air, or when the atmospheric pressure is diminished. This salt is also probably the agent by which the phosphate of lime in the blood is held in solution in a form in which it is not soluble in water, or in a solution of albumen. And thus we see that a most important office is performed by the inorganic elements of the blood, although their proportion is so small; and that without them its constitution would be incomplete.

When once the blood is formed, its growth and maintenance are effected by the constant repetition of the development of new portions. New materials are supplied to it by the lymph and chyle, and, by development, become like it, as its constituents are consumed in the repairs and secretions of the economy. By the stomach, the liver, and the other vascular glands, materials are prepared fit for the formation of blood ; and, by the excretory organs, the waste substance of the tissues—urea, carbonic acid, &c.—are carried out of the system, and its purity preserved. But not only so ; each part, by abstracting the materials it requires for its maintenance, is in the relation, as Treviranus remarked, of an excretory organ to all the rest. The muscles take the materials for their nutrition ; the bones remove their necessary aliment ; and thus neither fibrine, phosphate of lime, nor any other element is allowed to accumulate in excess.

Some impressive generalizations conclude this chapter, with which we close for the present our notice of this work, to resume it, however, in our next number. Thus, it appears that the development of the blood keeps pace with and is in proportion to the development of the race of beings. In the Invertebrata, the blood-corpuscles are on a par with the lymph-corpuscles of the oviparous Vertebrata. In fish, coincidently with the development of a brain and spinal cord, we find a larger quantity of blood. The fuller development of brain in reptiles is associated with a general further increase in the quantity and velocity of blood. As we rise in the scale of being we find a still further improvement in this fluid. In birds its velocity is greater than in reptiles ; the quantity of its fibrine is also augmented, its corpuscles are multiplied, and their size is diminished. And finally, in the Mammalia, the corpuscles appear developed in the highest form, corresponding with the superior development of the brain.

It is through the blood that the characters of the organic life are adjusted to the central nervous system. To its formation all the organs of that life minister, and it may therefore be regarded as the highest member of the parts concerned in the organic life, in the same sense as we regard the brain as the highest of the organs of animal life. "And this eminence of the blood is shown, first, by its chemical composition, which, as we have seen, is more highly organic than that of the greater part of the tissues; second, by the time at which it first appears in the embryo, in which, as the brain and spinal cord precede, in their rudiments, the other and subordinate organs of animal life, so the blood appears before any of the persistent organs of the organic life; third, by the complexity and number of the processes through which it is elaborated."

